# An Anaerobic-Type α-Ketoglutarate Ferredoxin Oxidoreductase Completes the Oxidative Tricarboxylic Acid Cycle of *Mycobacterium tuberculosis*

**DOI:** 10.1371/journal.ppat.1000662

**Published:** 2009-11-20

**Authors:** Anthony D. Baughn, Scott J. Garforth, Catherine Vilchèze, William R. Jacobs

**Affiliations:** 1 Howard Hughes Medical Institute, Albert Einstein College of Medicine, Bronx, New York, United States of America; 2 Department of Microbiology and Immunology, Albert Einstein College of Medicine, Bronx, New York, United States of America; Harvard School of Public Health, United States of America

## Abstract

Aerobic organisms have a tricarboxylic acid (TCA) cycle that is functionally distinct from those found in anaerobic organisms. Previous reports indicate that the aerobic pathogen *Mycobacterium tuberculosis* lacks detectable α-ketoglutarate (KG) dehydrogenase activity and drives a variant TCA cycle in which succinyl-CoA is replaced by succinic semialdehyde. Here, we show that *M. tuberculosis* expresses a CoA-dependent KG dehydrogenase activity, albeit one that is typically found in anaerobic bacteria. Unlike most enzymes of this family, the *M. tuberculosis* KG: ferredoxin oxidoreductase (KOR) is extremely stable under aerobic conditions. This activity is absent in a mutant strain deleted for genes encoding a previously uncharacterized oxidoreductase, and this strain is impaired for aerobic growth in the absence of sufficient amounts of CO_2_. Interestingly, inhibition of the glyoxylate shunt or exclusion of exogenous fatty acids alleviates this growth defect, indicating the presence of an alternate pathway that operates in the absence of β-oxidation. Simultaneous disruption of KOR and the first enzyme of the succinic semialdehyde pathway (KG decarboxylase; KGD) results in strict dependence upon the glyoxylate shunt for growth, demonstrating that KG decarboxylase is also functional in *M. tuberculosis* intermediary metabolism. These observations demonstrate that unlike most organisms *M. tuberculosis* utilizes two distinct TCA pathways from KG, one that functions concurrently with β-oxidation (KOR-dependent), and one that functions in the absence of β-oxidation (KGD-dependent). As these pathways are regulated by metabolic cues, we predict that their differential utilization provides an advantage for growth in different environments within the host.

## Author Summary

Knowledge of the basic biology of *Mycobacterium tuberculosis* is essential to identifying novel ways to combat the emerging threat of drug-resistant tuberculosis. Since the tricarboxylic acid (TCA) cycle is a cornerstone of metabolism and *M. tuberculosis* does not possess a “typical” TCA cycle enzyme set, much effort has been focused on elucidating the components of this pathway. Previous reports indicate that *M. tuberculosis* possesses a variant TCA cycle in which succinic semialdehyde replaces succinyl-CoA. Since this pathway does not conserve as much metabolic energy as the canonical pathway, we considered an alternative hypothesis: that *M. tuberculosis* might possess an anaerobic type α-ketoglutarate dehydrogenase. In this manuscript, we investigate this previously unknown activity for mycobacteria using a combination of genetic and biochemical approaches, and demonstrate that *M. tuberculosis* is capable of driving a conventional TCA cycle in an unconventional way. We also validate the existence of the previously described variant pathway and provide evidence that these two pathways are differentially utilized in response to a metabolic signal, fatty acid catabolism.

## Introduction

Despite the identification of *Mycobacterium tuberculosis* as the causative agent of tuberculosis (TB) over 125 years ago, two billion people worldwide are infected with this potentially lethal pathogen [1]. Each year, nearly ten million individuals will develop active TB; of these, approximately two million will die. Efforts to control the TB pandemic are now being threatened by the increasing prevalence of *M. tuberculosis* strains that are resistant to many or all available antimycobacterial drugs [2]. Understanding the biology of *M. tuberculosis* will facilitate the identification of targets for novel therapeutic approaches to preempt this persistent pathogen.

Determination of the full genome sequence of *M. tuberculosis* has enabled the prediction and assembly of conserved metabolic networks [3]–[5]. While such models are valuable for understanding the metabolic architecture of an organism, discrepancies between genome-based predictions and data from genetic and biochemical analyses occasionally arise. For example, of the ten *M. tuberculosis* genes predicted to encode subunits for α-ketoglutarate (KG) and pyruvate dehydrogenases, only two have been shown to possess the corresponding function [6],[7]. Indeed, biochemical surveys of enzymes of the tricarboxylic acid (TCA) cycle indicate that *M. tuberculosis* does not utilize a conventional KG dehydrogenase [8]. This disjunction at the conversion of KG to succinyl-CoA suggests either that this activity is non-essential for cellular metabolism, or that conversion of KG proceeds by means of a novel pathway. In support of the latter, it was recently shown that *M. tuberculosis* encodes enzymes capable of catalyzing a variant TCA cycle which uses succinic semialdehyde (SSA) rather than succinyl-CoA [8].

In this novel cycle, KG decarboxylase (KGD) catalyzes the thiamine pyrophosphate (TPP) dependent decarboxylation of KG to form SSA [8]. Subsequently, SSA dehydrogenase oxidizes SSA to succinate with the reduction of NADP^+^ to NADPH [8]. Similar to the canonical cycle, this cycle enables the extraction of reducing power to drive reductive processes, while still directing KG to succinate. However, similar to the glyoxylate shunt, this pathway bypasses the synthesis of ATP via succinate thiokinase. This bypass requires that pools of succinyl-CoA for synthesis of methionine, diaminopimelate, sulfolipids and heme be derived in an energy-dependent manner, either from succinate at the expense of ATP or from methylmalonyl-CoA via methylmalonyl-CoA mutase [9]. Despite this apparent inefficiency, KGD is predicted to play an important role in growth of the Mycobacteria on carbohydrates as the sole carbon and energy source [10].

In most aerobic organisms, the unidirectional oxidative decarboxylation of KG to succinyl-CoA is catalyzed by a ternary complex consisting of dihydrolipoyl dehydrogenase, dihydrolipoyllysine-residue succinyltransferase, and succinyl-transferring KG dehydrogenase. Interestingly, microaerophilic and strictly anaerobic organisms often utilize an alternative enzyme, KG: ferredoxin oxidoreductase (KOR), which can couple the interconversion of KG and succinyl-CoA to the reduction/oxidation of ferredoxin. KOR and other α-ketoic acid: ferredoxin oxidoreductase family members are typically composed of a CoA-coordinating α/γ subunit, and a TPP and iron-sulfur cluster containing β-subunit [11]. Measurement of this activity requires anaerobic conditions, both because these enzymes are irreversibly inactivated by O_2_, and because the commonly used chromogenic reporter substrate for the assay is rapidly oxidized under aerobic conditions [12]–[14]. These oxidoreductases have been identified in anaerobes and microaerophiles belonging to all three domains of life [15], suggesting their presence in the last universal common ancestor. In most cases, KOR is utilized for the reductive carboxylation of succinyl-CoA to KG [16],[17]. Yet, it has been suggested that the hyperthermophilic anaerobe *Thermococcus litoralis* might utilize KOR for the generation of succinyl-CoA to support biosynthetic reactions [13].

Here, we demonstrate that while *M. tuberculosis* can drive a TCA cycle with the canonical intermediates, it does so in an unconventional way using an anaerobic-type KOR. As homologs of KOR are broadly distributed throughout the Actinobacterial class, with the exception of the Corynebacterial and Bifidobacterial families, this enzyme likely plays a greater role in oxidative metabolism than was previously thought. In addition, we find that KOR is dispensable for growth of *M. tuberculosis* under conditions that promote the utilization of the variant SSA-containing TCA cycle, revealing that these cycles are regulated by different environmental cues. These studies indicate that the KOR pathway operates concurrently with β-oxidation, while the KGD pathway operates under conditions that do not favor fatty acid catabolism. These pathways likely endow *M. tuberculosis* with metabolic plasticity required for growth on diverse host-derived carbon and energy sources. Since a growing body of evidence indicates that lipids (for example cholesterol and fatty acids) are a predominant growth substrate for *M. tuberculosis* during infection [18]–[22], we speculate that flux through KOR represents an important step in intermediary metabolism *in vivo*.

## Results

### *Mycobacterium tuberculosis* encodes an anaerobic-type α-ketoglutarate: ferredoxin oxidoreductase homolog

**Figure 1:**
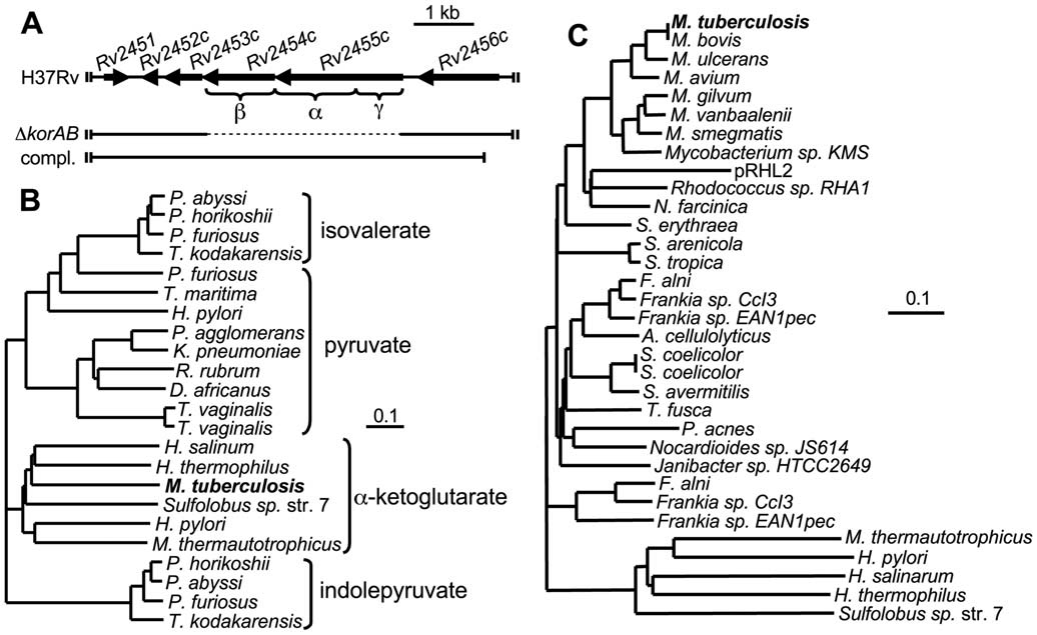
*M. tuberculosis* encodes an α-ketoglutarate: ferredoxin oxidoreductase. (A) Genetic map of the *M. tuberculosis Rv2454c*-*Rv2455c* (*korAB*) region. Conserved γ, α and β domains are indicated by brackets. The bar labeled Δ*korAB* denotes the region that was replaced by a hygromycin cassette using specialized transduction. The bar labeled compl. represents the region of the genome that was used for complementation of the Δ*korAB* strain. (B) Phylogenetic tree of the α subunits of characterized members of the α-ketoic acid: ferredoxin oxidoreductase family. Sequences were acquired from the NCBI protein database (www.ncbi.nlm.nih.gov). Alignments were made by the ClustalW method, trees were reconstructed by the Neighbor Joining method using the European Bioinformatics Institute server (www.ebi.ac.uk/Tools/clustalw2/index.html), graphics were generated using TreeView X (darwin.zoology.gla.ac.uk/~rpage/treeviewx/). α-ketoic acid substrates utilized by members of each clade are indicated to the right. The scale represents substitutions per residue. (C) Phylogenetic tree of α subunits of the α-ketoic acid: ferredoxin oxidoreductase found in several Actinobacteria. Alignments and trees were generated as described in B.

While all other activities of the TCA cycle have been measured from cellular extracts of *M. tuberculosis*, detection of KG dehydrogenase has remained elusive [6],[8]. Several anaerobic and microaerophilic organisms encode various evolutionarily related ferredoxin-dependent oxidoreductases that can interconvert specific acyl-CoA thioesters and their cognate α-ketoic acids, such as KG, pyruvate, indolepyruvate and isovalerate [23]. These oxidoreductases are of distinct ancestry from aerobic-type α-ketoic acid oxidoreductases such as pyruvate dehydrogenase and KG dehydrogenase [15]. Surprisingly, while *M. tuberculosis* does not express measurable KG dehydrogenase activity [6], it does encode a putative anaerobic-type α-ketoic acid: ferredoxin oxidoreductase (Figure 1A). Products of this locus include a fused α- and γ-subunit encoded by *Rv2455c* which shows a conserved binding site for coordination of CoA (GXXGXG), and a β-subunit encoded by *Rv2454c* which shows the highly conserved motif involved in TPP and iron-sulfur cluster binding (CXGCGX_n_GDGX_n_C) [11]. Based on intergenic distance and an extensive assessment of correlative expression data, *Rv2454c* and *Rv2455c* are likely organized in an operon with *Rv2452c* and *Rv2453c* [24]. While *Rv2452c* is of unknown function, *Rv2453c* encodes a putative molybdopterin-type dinucleotide biosynthesis protein.

It was not possible to predict the substrate for this oxidoreductase based on sequence homology alone, thus, α subunits of several biochemically characterized α-ketoic acid: ferredoxin oxidoreductases were aligned to reconstruct a phylogenetic tree (Figure 1B). In most cases, the resulting clades coincided with the preferred α-ketoic acid substrate. The *M. tuberculosis* homolog grouped within the clade for KG oxidoreductases (KOR). Further, this gene cluster was identified in 23 out of 43 individual Actinobacterial species for which the complete genome sequences were available. A phylogram derived from alignment of these homologs revealed a cladotypic pattern (Figure 1C) suggesting that the locus was present in the Actinobacterial ancestor. It is interesting to note that these genes were not identified in Bifidobacterial and Corynebacterial species. Indeed, *C. glutamicum* has been show to express a *bona fide* aerobic-type KG dehydrogenase, the sequence of which is highly similar to KGD of *M. tuberculosis* [25].

### *Rv2454c*-*Rv2455c* encode an aerotolerant coenzyme A-dependent KG oxidase activity

French pressure cell lysates prepared from aerobic cultures of *M. tuberculosis*, *M. bovis* BCG and *M. smegmatis* were assessed for methylviologen (MV, an artificial chromogenic electron acceptor) reductase activity using various electron donors. Enzymatic assays were performed under anaerobic conditions to prevent potential oxidation of the KOR complex, and to prevent the reoxidation of MV by O_2_ and components of the aerobic respiratory chain. Consistent with the presence of an anaerobic-type KOR, KG served as an electron donor for reduction of MV (Table 1). This activity was dependent on the presence of CoA and Mg^2+^ (data not shown). Unlike some α-ketoic acid: ferredoxin oxidoreductases, the mycobacterial activity was not stimulated by addition of TPP to the reaction mixture. However, a CoA-independent KG dehydrogenase activity was observed in the presence of TPP (data not shown), consistent with the previous report of KGD [8].

**Table 1:** CoA-dependent α-ketoglutarate: MV oxidoreductase activity in *M. tuberculosis*, *M. bovis* and *M. smegmatis*.

Strain	nmol MV_red_ min^−1^ mg^−1^ protein		
	KG	NADH	Pyruvate
*M. tuberculosis* mc^2^7000 (wild type)	1.0±0.1	1.7±0.1	<0.01
*M. tuberculosis* mc^2^7010 (Δ*korAB*)	<0.01	1.9±0.1	NM*
*M. tuberculosis* mc^2^7011 (Δ*korAB* - complemented)	1.3±0.1	1.9±0.1	NM
*M. tuberculosis* H37Ra	0.68±0.15	NM	NM
*M. bovis* BCG	1.6±0.2	NM	<0.01
*M. smegmatis* mc^2^155	1.7±0.2	2.9±0.2	0.59±0.02 (0.32)†

*: Not measured.

**†:** A fraction of pyruvate: MV oxidoreductase activity from *M. smegmatis* mc^2^155 was CoA-independent (shown in parentheses).

**Figure 2:**
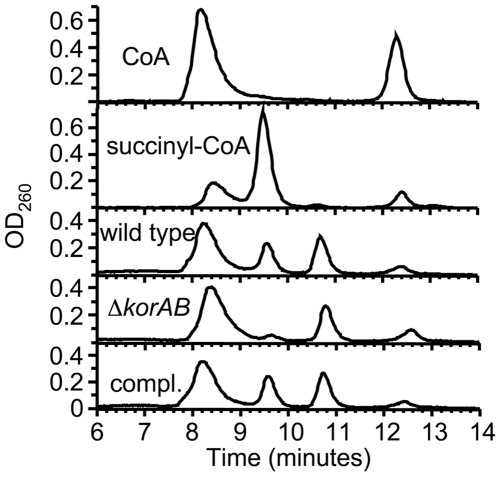
*korAB* is essential for the formation of succinyl-CoA from α-ketoglutarate and CoA by *M. tuberculosis*. Reaction mixtures containing CoA, KG, MV, MgCl_2_ and cell extracts from *M. tuberculosis* mc^2^7000 (wild type), the Δ*korAB* strain, and the complemented strain (compl.) were separated by ion exchange chromatography and CoA species were detected by UV absorbance (260 nm) following elution. CoA and succinyl-CoA were run as standards.

To determine whether the KOR product was succinyl-CoA, the reaction mixture was separated by ion exchange chromatography and eluted material was analyzed by UV absorbance at 260 nm. The absorbance profile of the eluted material was compared to those of CoA and succinyl-CoA standards. As shown in Figure 2, the reaction mixture prepared using cell extract from *M. tuberculosis* strain mc^2^7000 (H37Rv Δ*panCD* Δ*RD1* [26]) revealed a peak that coincided with that of succinyl-CoA, indicating that a significant fraction of CoA was activated to succinyl-CoA in the reaction mixture.

Assays in which pyruvate (Table 1), glyoxylate, oxaloacetate and 3-indole pyruvate (data not shown) were used as electron donors did not yield measurable MV reduction, indicating that the observed α-ketoic acid oxidation is likely specific to KG in *M. tuberculosis*. In contrast, *M. smegmatis* extracts could catalyze pyruvate-dependent reduction of MV (Table 1). Yet, a large fraction of this activity was found to be CoA-independent (Table 1). Unlike *M. tuberculosis* and *M. bovis*, *M. smegmatis* encodes an additional α-ketoic acid: ferredoxin oxidoreductase homolog, which is likely responsible for this activity. In addition, there was no measurable reduction of other electron carriers, such as NAD^+^, NADP^+^, FMN, FAD or menadione, when KG was used as an electron donor (data not shown).

**Figure 3:**
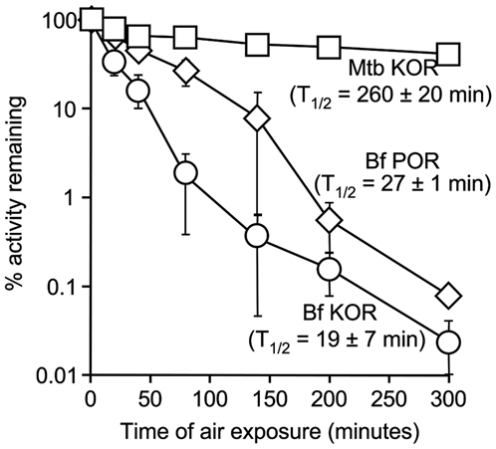
*M. tuberculosis* KOR activity is tolerant to O_2_ exposure. Whole cell lysates of *M. tuberculosis* and *B. fragilis* were exposed to air at room temperature and remaining KOR (*M. tuberculosis*, squares; *B. fragilis*, circles) and POR (*B. fragilis*, diamonds) activities were assessed under anaerobic conditions. Percent activity remaining was calculated by dividing the rate of methyl viologen reduction at time_x_ by that at time_0_ (% activity remaining  = rate_t = x_/rate_t = 0_×100). Data shown represent the mean ± standard deviation of three independent determinations.

Due to the presence of a solvent exposed iron-sulfur cluster, most α-ketoic acid: ferredoxin oxidoreductases are rapidly inactivated when exposed to O_2_ [13],[14]. Thus, the utility of these enzymes is usually restricted to anaerobic and microaerophilic environments. To determine whether the *M. tuberculosis* KOR was tolerant to air exposure, cell extracts were incubated under a normal atmosphere at room temperature. At various intervals the extracts were assayed for remaining KOR activity. As controls, air-exposed *B. fragilis* extracts were assayed for pyruvate: ferredoxin oxidoreductase (POR) and KOR. Similar to that which has been described for *B. thetaiotaomicron*, the *B. fragilis* POR was rapidly inactivated following air exposure (Figure 3) [14], as was the *B. fragilis* KOR (Figure 3). In contrast, when *M. tuberculosis* lysates were exposed to air the KOR activity was remarkably stable (Figure 3), indicating that *M. tuberculosis* KOR remains functional under aerobic conditions.

To determine the contribution of *Rv2454c*-*Rv2455c* (herein referred to as *korAB*) to KOR activity, the respective genes were deleted using specialized transduction [27]. Consistent with a previous study of gene essentiality in *M. tuberculosis*, mutations in *Rv2454c*-*Rv2455c* could be tolerated [28]. Codon 50 of *korA* through codon 334 of *korB* were replaced with a hygromycin resistance cassette (Figure 1A). While cell extracts from the resulting strain had wild type levels of NADH: MV oxidoreductase activity, there was no measurable reduction of MV using KG as an electron donor (Table 1) and succinyl-CoA production was diminished to <5% of that from the wild type extract (Figure 2). Importantly, introduction of an intact copy of *korAB* restored both KOR activity and succinyl-CoA production (Table 1, Figure 2). These results demonstrate that the *M. tuberculosis korAB* gene cluster codes for a KOR that is expressed and stable under fully aerobic conditions.

### KOR is conditionally essential for growth of *M. tuberculosis*

**Figure 4:**
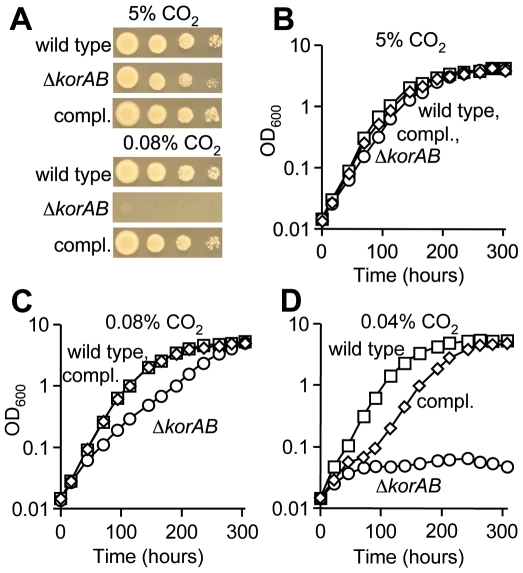
*korAB* is essential for growth of *M. tuberculosis* in the absence of sufficient levels of CO_2_. (A) Serial dilutions of *M. tuberculosis* mc^2^7000 (wild type), Δ*korAB* and the complemented strain (compl.) were spotted on supplemented 7H10 medium containing glycerol (0.5%), dextrose (0.2%), oleic acid (60 nl ml^−1^) and Tween 80 (0.05%). Plates were incubated under atmospheres with indicated amounts of CO_2_ for 20 days. (B–D) Growth of *M. tuberculosis* mc^2^7000 (squares), Δ*korAB* (circles) and complemented strain (diamonds) in supplemented 7H9 medium containing glycerol (0.5%), dextrose (0.2%), oleic acid (60 nl ml^−1^) and Tween 80 (0.05%) under an atmosphere containing 0.04% CO_2_ (B), 0.08% CO_2_ (C) or 5% CO_2_ (D).

Based on the lack of detectable KG dehydrogenase activity [6] and the presence of KGD [8], it has been proposed that *M. tuberculosis* catalyzes a variant TCA cycle in which succinyl-CoA is replaced by SSA [8]. However, as KOR is active in aerobically grown *M. tuberculosis*, it is possible that this enzyme can also functionally replace KG dehydrogenase in the TCA cycle. Consistent with a role for KOR in oxidative metabolism in *M. tuberculosis*, the Δ*korAB* strain was incapable of growth on solid medium unless the atmosphere was supplemented with abundant CO_2_ (Figure 4A). Indoor ambient CO_2_ levels were found to range from 0.078% to 0.084% during the course of these experiments. This CO_2_-dependent phenotype was also observed for *M. tuberculosis* strains H37Ra, CDC1551 and *M. bovis* BCG in which *korAB* was deleted (data not shown). In liquid medium, growth of the KOR-deficient strain was similar to that of the wild type strain when abundant CO_2_ was supplied (Figure 4B), whereas this strain was retarded under ambient air (Figure 4C) and fully inhibited upon further CO_2_ restriction (Figure 4D). Importantly, introduction of a cosmid containing *Rv2425c-Rv2456c* abolished this CO_2_ dependency (Figure 4A, C, D). This graded response to CO_2_ indicates that KOR-dependent decarboxylation of KG is an important source of CO_2_ in *M. tuberculosis* metabolism. It is predicted that the KOR-deficient strain is capable of growth with a broken TCA cycle due to the presence of the glyoxylate shunt. While this mode bypasses the production of CO_2_, it permits the extraction of reducing equivalents and production of biosynthetic precursors from two carbon units that enter the cycle.

### KOR is dispensable for growth upon inhibition of the glyoxylate shunt

**Figure 5:**
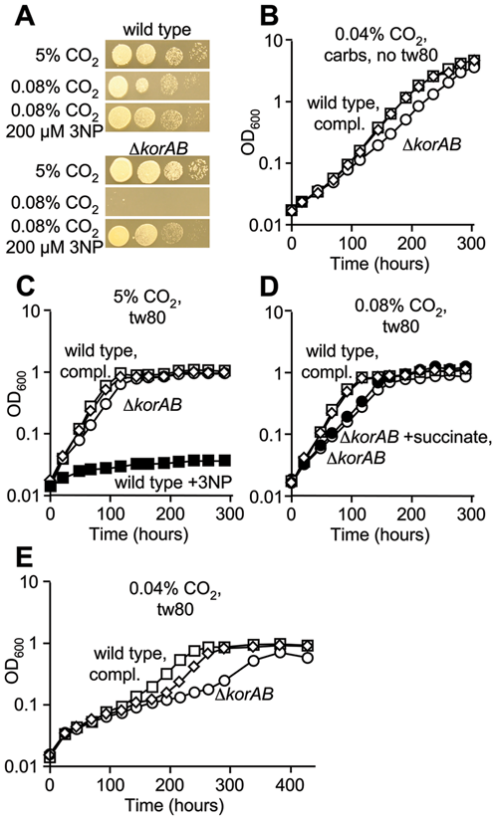
Conditional essentiality of *korAB* for growth of *M. tuberculosis*. (A) Serial dilutions of single cell suspensions of *M. tuberculosis* mc^2^7000 (wild type) and the Δ*korAB* strain were spotted on supplemented 7H10 medium containing glycerol (0.5%), dextrose (0.2%) and Tween 80 (0.05%) with or without 200 µM 3NP. Plates were incubated under atmospheres with indicated amounts of CO_2_ for 20 days. (B) Growth of *M. tuberculosis* mc^2^7000 (squares), Δ*korAB* (circles) and complemented strain (diamonds) in supplemented 7H9 medium with glycerol and dextrose without oleic acid or Tween 80 under an atmosphere containing 0.04% CO_2_. (C–E) Growth of *M. tuberculosis* mc^2^7000 (squares), Δ*korAB* (circles; filled circles, 0.1% succinate) and complemented strain (diamonds) in supplemented 7H9 medium with Tween 80 (0.5%) as the sole carbon source under atmospheres containing 5% (C), 0.08% (D), and 0.04% CO_2_ (E).

To determine whether the glyoxylate shunt is essential for growth in the absence of KOR, strains were plated on medium containing the isocitrate lyase (ICL) inhibitor 3-nitropropionate (3NP) [21],[29]. Surprisingly, 3NP was found to alleviate the CO_2_ requirement of the KOR mutant strain (Figure 5A). Moreover, this heightened CO_2_ dependency was also diminished by exclusion of fatty acids, namely oleic acid and Tween 80 (an oleic acid-polyethylene ester used to prevent cell aggregation), which are standard components of mycobacterial growth media (Figure 5B). Thus, while KOR is important for CO_2_ metabolism in the presence of exogenously supplied fatty acids, suppression of fatty acid utilization appears to promote activity of a compensatory pathway. As glyoxylate, a product of ICL, can inhibit SSA dehydrogenase [30], it is possible that the variant TCA cycle proposed by Tian *et al* [8] is favored under conditions in which catabolism of exogenous fatty acids is dampened.

To determine whether KOR is essential for growth of *M. tuberculosis* on fatty acids as the sole carbon source, the Δ*korAB* mutant was grown in medium containing Tween 80, which can be hydrolyzed by mycobacteria to form oleic acid and an inert non-metabolizable ethylene polymer. Similar to that which was observed for growth on mixed carbon sources, the Δ*korAB* mutant grew nearly as well as the wild type strain on Tween 80 when the atmosphere was supplemented with 5% CO_2_ (Figure 5C). As expected, 3NP inhibited growth of the wild type strain, indicating that the glyoxylate shunt is essential for growth on this source of oleic acid. Further, when the Δ*korAB* strain was grown under an atmosphere with ambient levels of CO_2_, there was a modest growth defect that could not be reversed by supplementation with succinate (Figure 5D). Unlike that which was observed with mixed substrates, the Δ*korAB* strain grew poorly under further CO_2_ restriction (Figure 5E), suggesting that gluconeogenesis might provide enough additional CO_2_ to support growth in the absence of KOR.

### Differential utilization of the KOR and KGD pathways

**Figure 6:**
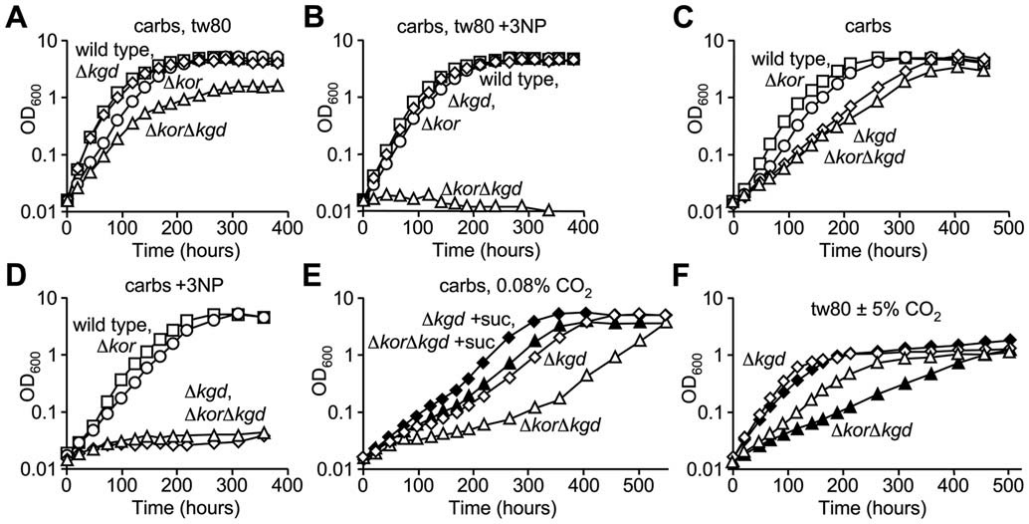
KGD and KOR are differentially required for growth of *M. tuberculosis*. *M. tuberculosis* strains mc^2^7000 (wild type, squares), Δ*korAB* (circles), Δ*kgd* (diamonds) and Δ*korAB* Δ*kgd* (triangles) were grown under a 5% CO_2_ (A–D) or 0.08% CO_2_ (E) atmosphere in supplemented 7H9 medium containing glycerol and dextrose (carbs, A–E) with 0.05% Tween 80 (A & B) or tyloxapol (C–E). 200 µM 3-nitropropionate (B & D) and 0.1% succinate (E, solid symbols) were added to the growth media. (F) Strains *M. tuberculosis* Δ*kgd* (diamonds) and Δ*korAB* Δ*kgd* (triangles) were grown under a 5% CO_2_ (open symbols) or 0.08% CO_2_ (solid symbols) atmosphere in supplemented 7H9 medium containing 0.5% Tween 80 as the sole carbon source.

To determine whether KGD is important for intermediary metabolism, *kgd* (*Rv1248c*) was deleted in *M. tuberculosis* mc^2^7000 and in the KOR deficient strain. Δ*Rv1248c* mutants were readily obtained despite the prediction that *Rv1248c* is essential for growth of *M. tuberculosis* on standard medium [31]. In medium containing both carbohydrates (dextrose and glycerol) and fatty acids (Tween 80), under a CO_2_ enriched atmosphere, growth of the Δ*kgd* strain was indistinguishable from that of the wild type strain regardless of the presence of 3NP (Figure 6A, B). Thus, under these conditions KOR is sufficient to maintain flux through the TCA cycle. However, the Δ*korAB* Δ*kgd* strain showed a slower growth rate relative to the wild type and single mutant strains (Figure 6A), indicating that either pathway can function to some degree in the *M. tuberculosis* TCA cycle. As growth of the Δ*korAB* Δ*kgd* strain was fully inhibited by the presence of 3NP (Figure 6B), blockade of all three pathways results in arrest of intermediary metabolism.

When the Δ*kgd* strain was cultivated in medium containing carbohydrates as the sole carbon source in the presence of 5% CO_2_, there was a marked defect compared to the wild type and Δ*korAB* strains (Figure 6C). Interestingly, growth of the Δ*korAB* Δ*kgd* strain was similar to that of the Δ*kgd* single mutant (Figure 6C), indicating that KOR contributes minimally during growth on carbohydrates as the sole carbon source. This notion is further supported by the observation that growth of the Δ*kgd* and Δ*korAB* Δ*kgd* strains was fully inhibited by the presence of 3NP, whereas growth of the wild type and Δ*korAB* strains was unaffected (Figure 6D). The growth defects observed for both the Δ*kgd* and Δ*korAB* Δ*kgd* strains were exacerbated by incubation under an atmosphere with an ambient level of CO_2_ (Figure 6E). As the growth defect of the Δ*korAB* Δ*kgd* strain was more severe than that of Δ*kgd* alone, KOR appears to have a minimal contribution to intermediary metabolism under these conditions. Growth of these strains was markedly improved by supplementation with succinate (Figure 6E), indicating that the growth defects of these strains are due both to limitations in generation of succinate and CO_2_. These observations indicate that KGD plays a predominant role in growth on carbohydrates as the sole carbon source.

Growth of the Δ*kgd* strain on Tween 80 as the sole carbon source was similar to that of the wild type strain regardless of the presence of CO_2_ (Figure 6F), consistent with a primary role for KOR in the TCA cycle under conditions that favor β-oxidation. In contrast, the Δ*korAB* Δ*kgd* strain was significantly more retarded for growth on Tween 80 than was either Δ*korAB* or Δ*kgd* alone (Figure 5C, 6F). Thus, while KOR is the major mediator for conversion of α-ketoglutarate during growth on fatty acids, KGD can also contribute to a minimal degree.

## Discussion

**Figure 7:**
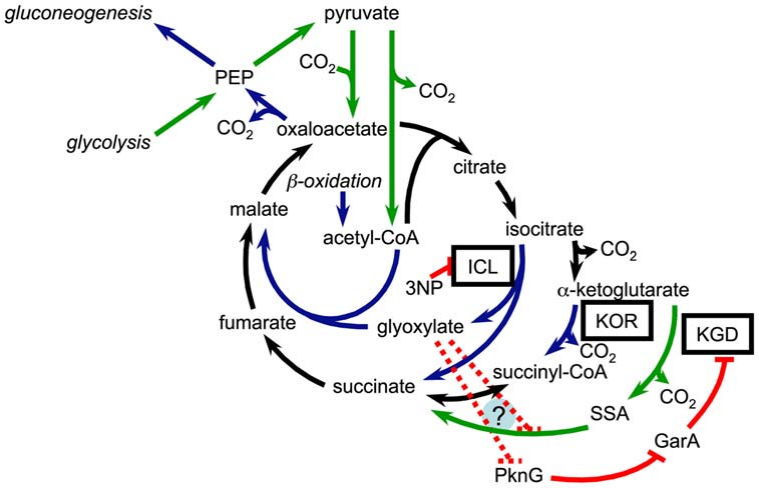
Integrated model of routes and regulation in the *M. tuberculosis* TCA cycle. The glyoxylate cycle (inner cycle), canonical TCA cycle (medial cycle), and variant TCA cycle (outer cycle) are depicted. Blue lines indicate pathways that are utilized concurrently with β-oxidation, green lines indicate pathways that are utilized during growth on carbohydrates as the sole carbon source, and black lines indicate pathways that are common to both modes of growth. Red lines indicate blocks imposed by 3NP on isocitrate lyase (ICL), PknG on GarA, and GarA on KGD. The dotted red lines represent the putative blocks imposed by glyoxylate on SSA dehydrogenase and PknG.

A previous report indicates that *M. tuberculosis* lacks a canonical TCA cycle, as CoA-dependent KG dehydrogenase activity was undetectable in crude cellular extracts [6]. Until recently it was unclear whether mycobacteria require an intact TCA cycle as they can produce TCA cycle-derived biosynthetic precursors via the glyoxylate shunt [9], although doing so would require that succinyl-CoA be formed in an energy dependent manner. Since isocitrate lyase (ICL) is dispensable for growth on carbohydrates as a carbon source [20],[21], it is likely that a TCA cycle of some form exists in *M. tuberculosis*. Based on biochemical studies of KG decarboxylase (KGD) and SSA dehydrogenase, it was recently proposed that SSA replaces succinyl-CoA in the *M. tuberculosis* TCA cycle [8]. While this pathway should support growth on carbohydrates when the glyoxylate shunt is inoperable (Figure 7), it still requires that succinyl-CoA be produced by alternate means. In addition to enzymes of this alternate pathway, *M. tuberculosis* and other mycobacterial species encode an anaerobic-type α-ketoic acid: ferredoxin oxidoreductase homolog that is most closely related to those that interconvert KG and succinyl-CoA. Here, we demonstrate that *M. tuberculosis* contains a KOR activity that results in the formation of succinyl-CoA, and requires the *korAB* gene cluster. As we were unable to identify the physiologic electron acceptor for this enzyme, it is currently unclear how KOR feeds into the cellular reduction/oxidation pools. Yet, since the *M. tuberculosis* KOR α subunit contains two hydrophobic stretches (from amino acids 249–273 and 304–340), it is possible that the complex is membrane associated and is reoxidized following interaction with another membrane associated redox partner.

KOR was probably overlooked in previous studies because of the requirement for anaerobic assay conditions resulting from the use of an O_2_-reactive reporter dye. In contrast to similar enzymes from the obligate anaerobe *B. fragilis*, the *M. tuberculosis* KOR activity was stable during extensive air exposure. Interestingly, sequence of the predicted iron-sulfur cluster coordination site of the *M. tuberculosis* enzyme was similar to that of other O_2_-sensitive KORs and does not contain a recognizable stabilization domain found in some δ-proteobacterial PORs [32]. While it is possible that the mycobacterial KOR is intrinsically aerostable, it is also possible that there might be an unidentified stabilization partner.

Despite the peculiarity of finding such an oxidoreductase in an obligately aerobic organism, KOR is conserved in many other Actinobacteria. As this class of eubacteria includes both aerobes and anaerobes, it is possible that the Actinobacterial ancestor was a facultative anaerobe, and that more stringent O_2_ requirements may have arisen with divergence of the various clades. Indeed, mycobacteria possess a suite of genes commonly associated with anaerobic metabolism, such as an anaerobic-type ribonucleotide reductase [33] and a respiratory nitrate reductase [34]. Although conditions for anaerobic cultivation of mycobacteria have yet to be defined, it has recently been demonstrated that *M. tuberculosis* can grow under an atmosphere containing as little as 1.3% O_2_ when provided with supplemental CO_2_ [35]. Thus, dampening oxidative metabolism results in a CO_2_ deficit, and likely leads to defects in lipid, arginine, adenine and uracil biosynthesis [36].

Consistent with its role in oxidative metabolism, we find that KOR is essential for growth of *M. tuberculosis* under an ambient atmosphere where the CO_2_ concentration is relatively low. As outlined in Figure 7, KOR functions concurrently with the glyoxylate shunt, likely to provide both succinyl-CoA and CO_2_, as well as reducing equivalents. Interestingly, KOR is dispensable for growth when the glyoxylate shunt is inoperable. Further genetic analysis revealed that KGD is essential for this bypass, indicating that the SSA pathway operates under conditions where utilization of exogenous fatty acids is minimal (Figure 7). Along these lines, we find that while KGD plays a critical role during growth on carbohydrates, it contributes little during growth in medium containing fatty acids. These observations suggest the presence of a metabolic regulatory cascade that is responsive to β-oxidation.

It was recently shown that the forkhead-associated protein GarA inhibits KGD activity in *M. smegmatis* [10] and its homolog OdhI inhibits KG dehydrogenase in *Corynebacterium glutamicum* [25]. In both cases, the serine threonine kinase PknG was found to modulate GarA activity via phosphorylation, although the signal for this regulatory cascade has yet to be described. In *M. smegmatis*, constitutive inhibition of KGD via unphosphorylated GarA results in a profound growth defect on dextrose and glycerol [10], indicating that this decarboxylase is predominantly utilized for growth on carbohydrates. Based on these findings, we predict that glyoxylate serves as the metabolic trigger for inhibition of KGD, likely through inhibition of PknG-mediated GarA phosphorylation (Figure 7). Furthermore, since we find that KOR cannot compensate for the loss of KGD during growth on carbohydrates alone, KOR must be subject to some means of regulation that has yet to be identified.

While a comprehensive analysis of the genetic requirements for growth and survival of *M. tuberculosis* indicates that genes linked to carbohydrate metabolism might be important early during infection [22], a large body of evidence indicates that lipids represent the major carbon source for growth and persistence of *M. tuberculosis in vivo* [18]–[22]. When lipids are processed via β-oxidation pathway, they are broken down in a series of steps into acetate and propionate (reviewed in [37]). Studies of ICL (for acetate utilization) and methylcitrate lyase (MCL, for propionate utilization) suggest that fatty acids might be important lipidic carbon sources for *M. tuberculosis* during infection [20],[21]. Yet, it has recently been demonstrated that sterols, such as cholesterol, can be also be catabolized by *M. tuberculosis* both *in vitro* and *in vivo* [18],[19]. As cholesterol catabolism also results in the formation of propionate and acetate, ICL and MCL are predicted to be required for use of sterols as well [38]. Given the concurrent function of the KOR pathway with the glyoxylate shunt, we predict that flux of KG runs largely through KOR, rather than KGD, during growth *in vivo*. The availability of these mutant strains will allow us to distinguish between these possibilities.

## Materials and Methods

### Bacterial strains and growth conditions

*M. tuberculosis* strains used in this study (Table 2) were derived from strain H37Rv and were routinely cultivated using Middlebrook 7H9 and 7H10 media (Difco, Sparks, MD) supplemented with NaCl (0.85 mg ml^−1^), oleic acid (60 nl ml^−1^), bovine albumin-fraction V (5 mg ml^−1^), dextrose (2 mg ml^−1^), and glycerol (5 mg ml^−1^). As indicated in the text, Tween 80 (0.5 mg ml^−1^ or 5 mg ml^−1^; a non-ionic surfactant) or tyloxapol (0.5 mg ml^−1^; a non-metabolizable non-ionic surfactant) were added to the growth medium. 100 µg ml^−1^ pantothenic acid was added for growth of pantothenic acid auxotrophic strains [26]. *M. smegmatis* strain mc^2^155 [39] was cultivated with 7H9 and 7H10 supplemented with dextrose (2 mg ml^−1^) and tyloxapol (0.5 mg ml^−1^ for liquid media). *E. coli* strain HB101, used for plasmid, cosmid and phasmid manipulation, was cultivated using LB medium. *B. fragilis* strain ATCC 25285 was cultivated using brain heart infusion medium supplemented with 5 µg ml^−1^ hemin and 5 mg ml^−1^ yeast extract [40], or using modified anaerobic minimal medium [41]. Bactoagar (1.5%) was added to media when necessary. 3-nitropriopionate (200 µM), carbenicillin (50 µg ml^−1^), hygromycin (50 µg ml^−1^ for *M. tuberculosis*, 150 µg ml^−1^ for *E. coli*) and kanamycin (20 µg ml^−1^ for *M. tuberculosis*, 40 µg ml^−1^ for *E. coli*) were added to the growth media as needed.

**Table 2:** *M. tuberculosis* strains and primers used in this study.

Strain	Genotype or relevant characteristic	Method of construction	Source
mc^2^7000	H37Rv Δ*panCD* Δ*RD1*	specialized transduction described in ref. 29	laboratory strain
mc^2^7010	mc^2^7000 Δ*korAB*	specialized transduction of strain mc^2^7000 with phage phAES*Rv2454c-5c*	this study
mc^2^7011	mc^2^7000 Δ*korAB attB*_L5_::pBH33K	electroporation of mc^2^7010 with cosmid containing *Rv2425c-Rv2456c*	this study
mc^2^7012	mc^2^7000 Δ*kgd*	specialized transduction of strain mc^2^7000 with phage phAES*Rv1248c*	this study
mc^2^7013	mc^2^7000 Δ*korAB* Δ*kgd*	specialized transduction of unmarked strain mc^2^7010 with phage phAES*Rv1248c*	this study
H37Ra	spontaneously attenuated derivative of H37Rv	spontaneous mutant	obtained from Wilbur Jones
mc^2^7014	H37Ra Δ*korAB*	specialized transduction of strain H37Ra with phage phAES*Rv2454c-5c*	this study

Primer	Sequence	Restriction enzyme used
Rv2455c-1	TTTTTTTTCCATAAATTGGGGCGTTGCACAATCGAGACGAAG	Van91I
Rv2455c-2	TTTTTTTTCCATTTCTTGGAGGTGAATCGGTCGCCGGTTAG	Van91I
Rv2454c-3	TTTTTTTTGCATAGATTGCCCGGCCAACCTACGATGATG	BstAPI
Rv2454c-4	TTTTTTTTGCATCTTTTGCATCAACCGCGCACCAGAGAC	BstAPI
Rv1248c-1	TTTTTTTTCCATAAATTGGGGCGTGCTGATCGCCTTTAGCCG	Van91I
Rv1248c-2	TTTTTTTTCCATTTCTTGGGCCGGGATCAAGCGAATCTCGCG	Van91I
Rv1248c-3	TTTTTTTTCCATAGATTGGTCGCGGAACTTGCGGTACATCTC	Van91I
Rv1248c-4	TTTTTTTTCCATCTTTTGGGATCCGCAAATGCAGCTGAGCC	Van91I

For growth experiments involving modified atmospheres, cultures were incubated in a controlled atmosphere chamber (Coy Laboratory Products, Grass Lake, MI) or in sealed 2.5 L AnaeroPack boxes (Mitsubishi Gas Chemical Co., Inc.). Atmospheric CO_2_ supplementation was regulated using an AC100 CO_2_ controller (Coy Laboratory Products). Atmospheric CO_2_ restriction was achieved by absorption with 40 ml of a 5% (w/v) solution of KOH [42]. Under such conditions, atmospheric CO_2_ was maintained at a level of 0.04%. Atmospheric CO_2_ determinations were made using a Bacharach model 2810 CO_2_ analyzer (New Kensington, PA), or a Gray Wolf DirectSense™ indoor air quality monitor (Trumbull, CT).

### Genetic manipulations and analysis

Purification of cosmids, plasmids and PCR products were performed using Qiagen products following the manufacturer's suggestions. Genetic manipulations of mycobacterial species were performed as described in [43]. *M. tuberculosis* strains mc^2^7010 (Δ*korAB*) and mc^2^7012 (Δ*kgd*) were constructed by allelic exchange using specialized transduction. The allelic exchange phasmids were constructed by amplifying 1 kb regions flanking *korAB* and *kgd* with primers described in Table 2. Purified DNA fragments were digested with indicated restriction enzymes. Fragments were ligated with Van91I fragments of p0004S (T. Hsu, unpublished) using T4 DNA ligase (NEB). The resulting allelic exchange substrates were digested with PacI, ligated to phAE159 [44] and packaged with MaxPlax packaging extract (Epicenter Biotechnologies) for propagation as shuttle phasmids in *E. coli*. Phasmids were electroporated into *M. smegmatis* mc^2^155 for phage propagation. Allelic exchange substrates were delivered to *M. tuberculosis* as previously described [27]. Mutant strains were confirmed by PCR. For construction of the double mutant strain, it was necessary to first resolve the hygromycin resistance cassette (which was flanked by γδ resolvase recognition sequences) in mc^2^7010 using the γδ resolvase containing vector pJH532. Cosmid pBH33K used for complementation of strain mc^2^7010 contains base pairs 2721932–2757635 of the *M. tuberculosis* H37Rv genome.

### Enzymatic assays

Enzymatic assays were performed using French pressure cell lysates prepared from mycobacteria grown in aerobic supplemented 7H9 medium, or from *B. fragilis* grown in modified anaerobic minimal medium. Cells were harvested by centrifugation at 3,500 X *g* for 10 min at 4°C. The following steps were performed in an anaerobic chamber (<1 ppm O_2_, Coy Laboratory Products) containing a gas mixture of 10% H_2_, 5% CO_2_ and 85% N_2_. Cells were washed in an equal volume of chilled anaerobic 100 mM sodium phosphate (pH 7.2), and resuspended in 0.1 volume chilled anaerobic phosphate buffer. Anaerobic phosphate buffer was prepared in the anaerobic chamber using distilled H_2_O that had been degassed by boiling immediately before introduction into the anaerobic atmosphere. Samples were removed from the chamber and cells were lysed under 70 kg cm^−2^ using a French pressure mini-cell and maintained under a stream of N_2_ to exclude O_2_. Extracts were clarified by centrifugation at 13,000 X *g* for 10 min at 4°C, flash frozen with liquid N_2_ and stored at −80°C until ready to use.

Reagents for enzymatic assays were purchased from Sigma-Aldrich and were dissolved in anaerobic phosphate buffer. Reaction mixtures were prepared under anaerobic conditions using anaerobic phosphate buffer containing 2.5 mM MgCl_2_ in QS-517-S quartz screw top cuvettes (Nova Biotech, El Cajon, CA). α-ketoic acid: MV oxidoreductase assays were performed as described [13], with the following modifications: 0.5 mM coenzyme A, 5 mM MV and 25 mM α-ketoic acid (pyruvate or KG) were added, and TPP was excluded. NADH: MV oxidoreductase activity was measured using 0.2 mM NADH and 2.5 mM MV. Reactions were started by addition of cell extract and MV reduction was measured spectrophotometrically at room temperature. Reduction values were based on an absorption coefficient of 12,000 M^−1^ cm^−1^ at 600 nm [45]. Protein estimations were made using the BioRad protein assay reagent.

For analysis of succinyl-CoA production, reaction mixtures were prepared as described above, however, 100 µM CoA and 50 mM MV were used. Reaction mixtures were passed through a 0.2 µm filter and incubated under anaerobic conditions for 30 minutes. Samples were stored at −80°C until time of analysis. CoA species were separated by ion exchange chromatography using an Äkta Explorer (Amersham Biosciences). CoA standards and reaction mixtures (500 µl) were injected onto a Mono Q HR 5/5 column in 50 mM potassium phosphate (pH 6.5) 50 mM NaCl. Nucleotides were eluted from the column with a linear gradient from 50 to 350 mM NaCl in 10 column volumes at a flow rate of 2 ml/min, and detected by UV absorbance (260 nm).

### Accession numbers

Sequences for phylogenetic analyses were acquired from the NCBI protein database (www.ncbi.nlm.nih.gov). Accession numbers corresponding to Figure 1B are as follows: *Pyrococcus abyssi*, NP_127041; *P. horikoshii*, NP_142630; *P. furiosus*, Q51801; *Thermococcus kodakarensis*, YP_184393; *P. furiosus*, Q51804; *Thermotoga maritima*, O05651; *Helicobacter pylori*, AAC38206; *Pantoea agglomerans*, X78558; *Klebsiella pneumoniae*, CAA31665; *Rhodospirillum rubrum*, X77515; *Desulfovibrio africanus*, CAA70873; *Trichomonas vaginalis*, U16822; *T. vaginalis*, U16823; *Halobacterium salinarum*, CAA45825; *Hydrogenobacter thermophilus*, BAB21494; *M. tuberculosis*, CAA16032; *Sulfolobus* sp., BAA10898; *H. pylori*, AAC38211; *Methanothermobacter thermautotrophicus*, NP_276168; *P. horikoshii*, NP_142702; *P. furiosus*, NP_578262; *P. abyssi*, NP_126972; *T. kodakarensis*, YP_182549. Those corresponding to Figure 1C are as follows: *M. tuberculosis*, CAA16032; *M. bovis*, NP_856129; *M. ulcerans*, ABL05848; *M. avium*, YP_880944; *M. gilvum*, ABP45093; *M. vanbaalenii*, ABM14742; *M. smegmatis*, YP_888909.1; *Mycobacterium* sp. KMS, YP_939624; pRHL2, *Rhodococcus jostii*, ABG94195; *Nocardia farcinica*, BAD58508; *Saccharopolyspora erythraea*, CAM03198; *Salinispora arenicola*, ABV96568; *S. tropica*, ABP53176; *F. alni*, CAJ59716; *Frankia* sp. CcI3, ABD09939; *Frankia* sp. EAN1pec, ABW15421; *Acidothermus cellulolyticus*, ABK52058; *Streptomyces coelicolor*, CAB60189; *S. coelicolor*, CAC08296; *S. avermitilis*, BAC72589; *Thermobifida fusca*, AAZ56707; *Propionibacterium acnes*, EEB67312; *Nocardioides* sp. JS614, ABL80079; *Janibacter* sp. HTCC2649, EAQ00280; *F. alni*, CAJ65422; *Frankia* sp. CcI3, ABD13820; *Frankia* sp. EAN1pec, ABW16201; *M. thermautotrophicus*, NP_276168; *H. pylori*, AAC38211; *H. salinarum*, CAA45825; *H. thermophilus*, BAB21494; *Sulfolobus* sp., BAA10898. *R. jostii* pRHA1 KorA was assembled from translated sequence derived from NC_008270.
